# Critical path of women in situations of intimate partner violence^1^

**DOI:** 10.1590/1518-8345.2414.3025

**Published:** 2018-08-09

**Authors:** Daniella Yamada Baragatti, Diene Monique Carlos, Maria Neto da Cruz Leitão, Maria das Graças Carvalho Ferriani, Eliete Maria Silva

**Affiliations:** 2 PhD, RN, Secretaria Municipal de Saúde, Prefeitura Municipal de Campinas, Campinas, SP, Brazil.; 3 Post-doctoral fellow, Escola de Enfermagem de Ribeirão Preto, Universidade de São Paulo, PAHO/WHO Collaborating Centre for Nursing Research Development, Ribeirão Preto, SP, Brazil.; 4 PhD, Professor, Escola Superior de Enfermagem de Coimbra, Coimbra, Portugal.; 5 PhD, Full Professor, Escola de Enfermagem de Ribeirão Preto, Universidade de São Paulo, PAHO/WHO Collaborating Centre for Nursing Research Development, Ribeirão Preto, SP, Brazil.; 6 PhD, Associate Professor, Faculdade de Enfermagem, Universidade Estadual de Campinas, Campinas, SP, Brazil.

**Keywords:** Intimate Partner Violence, Domestic Violence, Spouse Abuse, Gender-Based Violence, Help-Seeking Behavior, Qualitative Research

## Abstract

**Objective:**

To know and analyze the critical path followed by women subjected to intimate partner violence until reaching a Reference Center of a Brazilian city, from the perspective of these women.

**Method:**

Qualitative research, based on the concept of critical path. Participant observations of the support group of the Reference Center and interviews with ten women in situations of violence who were followed up in this service.

**Results:**

the motivating factors for women to seek help were the increase in the severity of the aggressions, the impact of the violence on their children and support from their family. They go to several sectors and services before reaching the Reference Center, such as the police and legal counsel, health and social services. They find little effective responses from the services, and care is indicated as the most relevant response factor to face the situation.

**Conclusion:**

the assistance is fragmented and dependent on the posture of the professional. The Reference Center is recognized as an important place of support for women. In addition to governmental efforts to maintain the network structured, the permanent education of the working professionals is also fundamental**.**

## Introduction

Violence against women is a violation of human rights and a serious public health problem in the contemporary world. Approximately one third (30%) of women who have been in a relationship experienced physical and/or sexual violence perpetrated by the intimate partner, and in some regions this rate reaches 38%^1^. A systematic review conducted with studies from 1990 to 2011 to estimate the global and regional prevalence of intimate partner homicide found that intimate partner was six times higher than the proportion of male homicides^2^.

Women who experience domestic violence have lower quality of life^3^. According to the literature, there are physiological mechanisms that associate exposure to violence with different health effects; therefore, there may be implications for mental health and cognitive functioning. In addition, the immune system may become weakened, increasing the risk of cancer and viral infections^4^ ^-^ ^5^.

The construction of care for this population must include the identification of opportunities to provide support and connect women with other services they need. To this end, it is important to know the itinerary of these women in order to identify the possible gaps and weaknesses in the services and in their connections, the potential of the care offered by the various services and sectors, and the challenges of this area, which is essential to the tertiary prevention of violence against women. The sequence of decisions and actions taken by an affected woman to address the violence she experienced and the responses found in her search for assistance is called a “critical path”, which describes the steps prior to arrival at the Reference Centers^6^. This concept proposed by the Pan American Health Organization (PAHO) is associated in other countries with the term “help-seeking behavior”.

The critical path is an interactive process comprising the factors that motivate or inhibit affected women and their actions and the social responses they find, which in turn have an impact on their path. These women’s decisions are influenced by their information, their knowledge, their perceptions and attitudes, the resources available, their previous experience, the importance given to the situation and support or obstacles encountered^6^.

The present study was based on the above and its main contribution is to give voice to women in situations of violence, seeking to understand the path they follow to obtain support to cope with this situation. This knowledge is well developed in international studies, but still incipient in the Brazilian context and in South American countries. More in-depth theoretical discussions are necessary to understand this phenomenon and expose possible cultural differences and less severe intimate partner violence^7^ ^-^ ^8^. Thus, the objective of this study was to know and analyze the critical path followed by women subjected to intimate partner violence until reaching a Reference Center of a Brazilian city, from the perspective of these women.

Considering the complexity of the problem, health services must provide a network of services to women in situations of violence, with the joint action of areas such as education, public security, social assistance and culture. Health care professionals must understand this in order to expand and qualify the care provided to these women and to address the difficulties in the management of domestic violence^9^. 

## Method

Qualitative, descriptive and exploratory research, based on the concept of critical path proposed by PAHO^6^. It was conducted in the Reference Center for Women in Situation of Violence located in Campinas, São Paulo, Brazil. 

The Reference Center offers individual assistance with a psychologist, a social worker and a lawyer, allows women to participate in workshops to learn activities such as hairdressing, and provides an appropriate place with a social educator to leave the children during care and a support group with weekly meetings, in addition to tours, and other activities. 

The participants of this study were women who sought care in this service between the years of 2013 and 2016, and who met the inclusion criteria: (I) being over 18 years old; (II) having suffered domestic violence perpetrated by an intimate partner of any nature; (III) having sought help at the Reference Center and (IV) continuing care by going at least a second time to the service.

The main researcher began to approach the service by attending the support group. At the end of the meetings, she presented the research proposal individually to the women participating. Those who agreed to participate were interviewed on pre-established days and times, depending on their schedule. 

Data collection took place between September 2015 and November 2016, through participant observation and semi-structured interviews. Participant observation was conducted during the activities of the support group of the Reference Center. The support group occurs once a week and lasts two hours. It is led by the psychologist of the service and the participants are women who seek the service for care. On average, 15 women participate in each group.

The main researcher participated in 15 different groups, totaling 30 hours of observation. After the meetings the notes were recorded in the field diary: the people present that day, which subjects were covered, which techniques were used, the main sentences said, the places they went for help, and impressions regarding the environment and possible complications. 

The interviews used as guide the instrument developed by PAHO to investigate the critical path of women in situations of violence, which was translated and validated by Brazilian researchers^6^ ^-^ ^8^. The script of the semi-structured interviews had the objective of identifying the path they followed when they decided to seek help, their perception of care and the aspects that facilitated or hindered the process of seeking help^8^. 

The interviews were individual and occurred in closed rooms in the premises of the Reference Center. The interview script began with questions addressing the socio-demographic characteristics of the women, such as age, civil status, level of education, number of children, place of residence, occupation and family income. Then the following open-ended questions were asked: “Can you talk about the situation of violence that you experience or experienced? Did you tell anyone? What motivated you to seek help and where did you go the first time?”. For each location where the women reported they had sought help, they were asked: “Who served you? How was the process? What did they say? How were you treated? Did they refer you to another service?”. 

The number of women interviewed was not previously established. During the interviews, as the content was repeated, that is, when the critical path followed by them was established by their speeches, the interviews were closed, totaling ten women. The number of interviews is also justified by data that was corroborated during the observations of the support group, in which the interviewees and other participants described their stories and, consequently, the critical path followed until reaching the support service. In qualitative research, the emphasis is on the experiences and understandings of people about particular phenomena, which can be understood even with a smaller number of participants^10^.

All interviews were recorded using a voice recording application for mobile phones and were later manually transcribed. The longest interview lasted one hour, 57 minutes and 15 seconds, while the shortest interview lasted 26 minutes and 41 seconds.

The data from the interviews and from the participant observations were analyzed using the inductive thematic analysis technique, a method used to identify and analyze patterns of qualitative data mainly related to the language used by the participants^10^. The following steps were followed: (I) familiarization with the data - after transcription of the interviews and systematization of the materials from the participant observation, an exhaustive reading of the collected material was done; (II) coding of the relevant information obtained from the data - pithy labels for important features of the data of relevance to the research, the so-called codes, were established according to the guiding questions, the research objective and the concept of critical route; (III) searching for coherent and meaningful themes or patterns in the relevant data - the codes created from the answers to the research questions were grouped in the themes “The beginning of the critical path: motivating factors”; and “Services on the critical path: actions taken and response factors”; (IV) reviewing themes - checking if the themes are in accordance with both the coded extracts and the full data-set. In order to ensure the validity of the analyzes, other researchers checked the codes and themes according to their reading of the transcribed material and verification of the audios of the interviews; (V) defining and naming themes - writing a detailed analysis of each theme, identifying its essential characteristics; (VI) final draft - final element of the analytic process that involves weaving together the analytic narrative and contextualizing it in relation to relevant literature, legal instruments and theoretical concepts.

The research followed ethical aspects of research involving human beings and was approved by the Research Ethics Committee of the School of Medical Sciences of the State University of Campinas (Protocol No. 1,073,136/2015) and by the public bodies related to Citizenship, Inclusion, Social Assistance and Health. It also obtained authorization from the coordination of the Reference Center. Secrecy of the participants was kept with the use of fictitious names, and their information was only collected after signing the Informed Consent Form. 

## Results

### The beginning of the critical path: motivating factors

The women decided to seek for help when the aggressions became more severe; when the situation became unsustainable or when there was some extreme situation, such as a daughter trying to commit suicide due to violence suffered by the mother: *(...)Then he attacked me and I said - No! Enough! 60, almost 61 years getting beaten up! (...)And also because I saw that it was getting very serious, you know? Because the way he attacked me, the next day I woke up with a swollen face, a swollen eye.* (Maria) *(...)The week that my daughter was having tests it seems that he got even angrier, started cursing me and we would not sleep, and it turned out that the children would not sleep either. My daughter did not do well on the tests. Then she went and took a bunch of pills a couple of days before Christmas.* (Fernanda)

According to their answers when asked if they told anyone about the situation of violence, the main support received was that of their children: *(...) No! You do not enter here. And leave, otherwise I’ll call the police! Then he left, went away. Then she said, “Mother, you’re not going to leave. You are staying here, in my house”. Then she took the key and ran to the other house to get clothes for me, you know? She ran there, got some clothes and went back to her house, where I stayed.* (Maria)

Other supports received by these women were from their mothers, friends and sisters. For some women, this support was perceived as the most important motivation for seeking help, along with the support of their children. *I tell it to my mother, always. Everything that happens to me, my mother knows.* (Ivone) *Yes, yes, I have a friend who participates a lot (...) So she knows what happens.* (Léia) *Then I said... then I told her... [the sister]. Then she said: no, I always knew it in my head; I knew those were not falls.* (Elaine).

On the other hand, children, mothers and family members are often not perceived by women as a source of support, since they do not identify the episodes reported as violent or they underestimate the situation: *(...) I told my mother about the fights, I told my brothers, I told them all, but they thought it was only a tiff, and that we would get back together. Then when they saw that it was not a silly thing, that I was beaten up... Well, it was too late and nobody could help me anymore.* (Camila)

### Services on the critical path: actions taken and response factors

Among the actions taken by these women, the main ones were filing a Police Report (PR); seeking Women’s Police Stations (DDMs) or conventional police stations; and seeking health and social care services. Throughout this process, they obtained different answers. The path of women in situations of violence is not linear. The services that composed the critical path of each woman and the service that referred them to the Reference Center are shown below, in Table 1.

**Table 1:** Services on the critical path of women and who referred them to the Reference Center. Campinas, SP, Brasil, 2016

Name	Services on the critical path	Service that referred them to the Reference Center
Nélia	RC*/DDM^†^	DDM^†^
Ivone	Judiciary/CRAS^‡^/RC*	RC*
Carol	Hospital/DDM^†^/Shelter	Shelter
Elaine	RC*/CRAS^‡^/Hospital/DDM^†^/Police	CRAS^‡^
Elisângela	CRAS^‡^/RC*/Hospital/Conventional Police Station	CRAS^‡^
Camila	Police/ Conventional Police Station /DDM^†^/Judiciary/RC*	DDM^†^
Fernanda	Hospital/NGO^§^	Hospital
Léia	Police/DDM^†^	DDM^†^
Maria	Judiciary/DDM^†^/Hospital/RC*	Public Defender

*RC - Reference Center; †DDM - Women’s Police Station; ‡CRAS - Social Assistance Reference Center; §NGO - Non-Governmental Organization.

Among the interviewees, eight filed a police report, usually more than once: *(...)I filed a report. Then he would get the subpoena and tear it apart. Then I would come back there again ... the subpoena came, he tore it again.* (Elaine). Five filed the report in the DDM: *(...)I went to the woman’s police station several times, to file a police report against him*. (Ivanete); two filed a report in both police stations (DDM and conventional): *(...) I’ve been to both* (Carol); and one filed the report only in the conventional police station: *(...) I’ve already been there [in the police station] because of a physical assault (...) And I haven’t been to the women’s police station*. (Elisângela). The women only went to the conventional police station on weekends, when the DDMs are closed. However, according to the speeches of these women, the police report did not do any good. One reason for not filing a report is because they did not believe it would help: (...) *Because when I got beaten up and I was all bruised, they said to me at the hospital, “look, let’s file a report”. I told her: a report for what? What are you going to do for me? I did not file the report, but I went to the police station.* (Carol).

Although DDMs are the service that most referred women to the Reference Center, it is one of the most criticized. In DDM they are constantly discouraged to continue the process, pressured to give their partner another chance, and treated with coldness and disregard: *(...) Then the DDM attendants said, “Try again. If he wants to assault you, you come back here”. (...) He lost control again. So I went back to DDM and they said, “Oh! What do you want? Do you want someone from here to come into your house with a straitjacket to bring him here? This is never going to happen”.* (Elaine)

There were women who went to the Conventional Police Department and evaluated the service positively: *(...) The attendant was a man and it was normal. He even oriented me, you know? He told me I should continue the process, because many cases start with an assault and then it can become something worse, such as death, right? The guy explained to me that what I had suffered was an assault. He told me to continue, to go to the DDM, he oriented me.* (Camila)

Another service criticized by women is the police. According to the data obtained, the police were called mostly at the time of a violence episode, but the response was generally not positive: *(...)Then the police officer said, “Look, m’am, we can’t do anything. He did not hit you, did not draw blood, so we cannot arrest him”, and it was always like that.* (Carol) 

Some women in situations of violence went to the judiciary with the main objective of separating from the violent husband. The evaluation of the service was negative most of the time, because they considered that they were pressured to reconcile with the partner: *(...) they told me, “No, it has to be like that, there must be a reconciliation. It can’t be like that, the first time and you already split up”. But then if a death occurs in the marriage, who is going to be accountable? Not them.* (Ivone)

Regarding the health services, there were reports of search for care in the Primary Health Care (PHC) unit - *(...) I went to the health center, talked to them and they told me to take him there.* (Maria) -, in the emergency unit - *(...) I went to the hospital one time when he cut open my head.* (Elisângela)- and in the specialties department of a hospital where her husband was followed up - *(...) he had tried to choke me on that day when I went there to see the [specialty] doctor. The doctor saw it and said, “We’re going to make an appointment for him right now”*. (Elaine). The reports also show that health professionals offer help to the husband-offender, both in the PHC and in the specialty service.

Regarding the effectiveness of the health services offered to women in situations of violence, it was observed that in hospitals and PHC units the referral to other care services, such as the Council of Guardianship and the Reference Center, is a common practice. Women consider themselves helped when they feel welcomed, have their complaints heard and, especially, when they are referred to another service: *(...) Fátima [Nurse] talked to me, tried to calm me down... I told her I was like that because of my husband, that he drank a lot and verbally abused me. She already knew me, she talked to me, tried to calm me down.* (Carol)

There were two women who evaluated the care received in the health units negatively. They affirmed that these services do not always have mental health professionals, such as psychologists and psychiatrists, which makes it difficult to provide care to women who are in a situation of violence and who do not fit into a profile for referral to other services that have these professionals. *Even there in the Health Center I cried, begged, and humiliated myself to be able to see a psychologist...* (Ivanete)

Women frequently sought social work for assistance, mainly in the Social Assistance Reference Center (CRAS). Many arrived at the Reference Center by referral from social workers not only from CRAS, but also from the shelter, the Public Defender’s Office and a Non-Governmental Organization (NGO): *(...) Then I went to the CRAS in the neighborhood where I live and I talked to the social worker. She soon referred me to a psychologist and to the Reference Center.* (Camila) *(...) It was in the shelter that the social worker sent me here, to the Reference Center.* (Carol) *(...) it was in the one [NGO] that I was. She told me, she wrote a letter and said on this day you take this paper to this place. Then I got it and I came here.* (Ivanete)

All the women evaluated the Reference Center positively, affirming that they feel good in the service and feel comfortable to forget about their problems a little, to cry or to say whatever they think without being judged; besides these aspects, they reported this was the most important support found in their paths: *(...) Here. After I started coming here, the girls gave me support, they talked to me. I am already changing; the girls can vouch for it. I want to change.* (Elaine) *(...)Let’s say that after I’m here in the Reference Center I’ve been able to filter things better, I’m much better.* (Léia)

## Discussion

**Figure 1:**
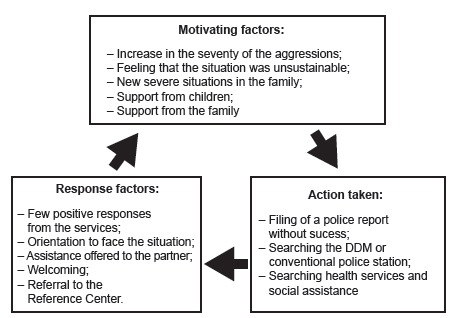
The critical path: schematic representation. Adapted from Shrader, Sagot (2000)^6^

The motivating factors, the actions taken and the response factors present in the critical path of the women participating in the study are represented in Figure 1: 

Regarding the motivating factors, the search for help also increased according the severity of the violence in researches in Canada, Serbia, Turkey, Mexico and Africa^11^ ^-^ ^15^. The results of a study conducted in Canada showed that even services such as shelters and victim assistance programs were sought by these women, indicating the increased risk of these situations^11^. The main reason women did not think about seeking formal support was because they did not consider the violence they suffered so serious and/or they thought that they would be able to deal with it alone^12^. The continued availability of a range of services that ensure the safety of these women and their families is necessary to meet their long-term health and economic needs^11^.

As in this research, studies conducted in Mexico, Africa and in the United States, found that the main support received by women in situations of violence was from their family^14^ ^-^ ^16^. Another Brazilian research also corroborates the importance of the family as a motivating factor, since most of the women did not seek formal support and only told their family about the situation of violence experienced^7^. The impact of violence on children has been an important trigger for women to seek help, corroborating a study in Serbia^12^; this aspect is relevant not only for women, but also for children and adolescents who witness violence. 

There were times when the family was not perceived as a source of support by the women interviewed. This was also found in a study with women in situations of violence in Turkey^13^. Given the importance that this non-formal support has for the management of this situation, this aspect is as a relevant obstacle in coping with the violence suffered and it can also increase the guilt felt by these women^13^.

The beginning of the critical path is when women decide to break the silence by revealing their situation to a person outside their immediate family and friends. The critical path describes the responses found by women throughout this process^6^. The DDMs are an important resource for women in situations of violence, and Brazil was a pioneer in the creation of this type of service. The professionals that work in the DDMs should be trained to provide better care and welcoming for women in situations of violence, mainly because it is the service that is closer to these women and which has the greater potential for this assistance; in addition, it is recognized and legitimized by the population. The results regarding the DDM in this study are similar to those found in southern Brazil, where most of the women sought this service and evaluated it negatively^8^. 

The only positive evaluation was of a conventional police station, not specialized in women, with the assistance conducted by a man. Considering the findings of this study and taking into account that in the DDM only women provide assistance, because this profile is expected to be better for other women, we question if what makes a difference are the attendants being women or professionals that were trained and participated in discussions on the problem, regardless of gender. 

As in this research, studies in Brazil, Turkey, Mexico and the United States found that the majority of women in situations of violence who sought formal help called the police^7^ ^,^ ^13^ ^,^ ^17^ ^-^ ^18^. In the southern region of Brazil, a study conducted to assess the critical path of women in situations of violence found that the police are unable to ensure the current protective measures^8^. 

The findings related to the judiciary are corroborated in the literature, which brings reports that workers in the legal sector pressure women to remain in the situation of violence^8^. This aspect also reinforces the gender stereotypes present in daily life.

All interviewees contacted health services for violence-related issues, a result similar to a research conducted in Canada, where 81.8% of women in situations of violence had contact with health services in the last year^19^. 

Still regarding the search for help in health services, studies emphasize that health professionals are the first ones to have contact with women in situations of violence, since they generally seek health care due to aggression injuries, even if they do not state the real reason for their bruises^4^ ^,^ ^11^. In this sense, the potential of these services, in particular the PHC, for the detection, reception and referral of cases of violence can be highlighted, since they are services that are close and available to the community.

Studies indicate that women seek health care without reporting the situation of violence^4^ ^,^ ^11^. However, in this study, the findings showed that they directly reported the situation, including psychological violence, in the primary care services. Emergency services were also sought for complaints of physical violence. Finally, the specialty service was sought by a woman because she frequently went there since her husband was being followed up, which demonstrates the importance of the bond of trust.

Another interesting finding is that health professionals offered assistance to the partner. The resolution of a situation of violence includes not only the follow-up and strengthening of women, but also the reception and treatment of male perpetrators. This discussion has been emphasized in recent literature^20^.

The negative evaluations of the health services in this research can be explained by the fact that health professionals have difficulties to deal with problems such as violence, because they do not consider this situation as part of their field of activity. This is also supported by other studies, including one conducted in the same city of the current research, which revealed that some professionals have difficulties to identify violence as an aspect associated with the search for the health service and demonstrate lack of preparation for care and referral of these cases^21^ ^-^ ^22^. 

Social services, such as the CRAS, were sought by the women interviewed. Again, the evaluation of the service is related to the way they were treated by the professional who attended them, and it is positive when they feel welcome and are referred to another service. According to a study carried out in the same city, assistance to violence is person-dependent^22^. In a Canadian study on delayed help-seeking in situations of violence, the authors described characteristics considered important in the professionals for women to reveal their situation, such as high level of trust, non-judgmental attitude, friendly listening posture and cross-cultural listening^19^. 

In the participant observations of the Support Group - one of the services offered by the Reference Center - this space was perceived as important for empowerment and relief of suffering. Thus, even if they have not left the situation of violence, the Reference Center is an important space for these women, where they can find understanding and non-judgmental attitudes. The recommendations of a study by English researchers in West Africa are that interventions related to women in situations of violence should focus more on prevention than on mechanisms for women to leave violent relationships, thus respecting their decisions^15^.

It was noticed that women seek several places before reaching specialized services with professionals trained to deal with cases of domestic violence against women. Thus, it is important to emphasize that professionals in the women’s care network must be constantly aware of the profile of this public and of the services offered in the Reference Center so that they can refer these women more properly, since not all of them are capable to do this. 

In addition to the training of professionals who work in the care network for women in situations of violence, the Reference Center must be increasingly disseminated by the media and community lectures must be held. If professionals do not have enough knowledge about the service, the situation is even more alarming compared to the general population. Experiences in Nicaragua, India and Uganda have demonstrated the importance of community-based actions to change gender and power stereotypes; these strategies are triggers for cultural, social and political changes in broader contexts^23^.

All the interviewees evaluated the Reference Center positively, perhaps because the subjects of this research were women who received assistance in this service, which is one of the limitations of this study. Another limitation is the lack of interviews with professionals who assist women in situations of violence, which could broaden the discussions.

## Conclusion

Despite the importance of a network of services for women in situations of violence, in practice, according to the discourses of the women interviewed, the service is fragmented. The Reference Center is considered a fundamental source of support for women in situations of violence, and it is important that the cities implement this service. In addition to the governmental efforts to maintain the network structured, the permanent education of the working professionals is also fundamental, since the positive evaluation of the service is related to the attitude of those who provide assistance and their knowledge on the services available. 

The referral by the different health units to other services, and especially to the Reference Center, should be preceded by a warm and ethic professional attitude with technical knowledge, aimed at effectively helping women in situations of violence. New studies with qualitative approaches to assess the vision of professionals regarding these experiences are recommended.
